# The Deficit of Multimodal Perception of Congruent and Non-Congruent Fearful Expressions in Patients with Schizophrenia: The ERP Study

**DOI:** 10.3390/brainsci11010096

**Published:** 2021-01-13

**Authors:** Galina V. Portnova, Aleksandra V. Maslennikova, Natalya V. Zakharova, Olga V. Martynova

**Affiliations:** 1 Institute of Higher Nervous Activity and Neurophysiology of RAS, 117485 Moscow, Russia; alexm2004@list.ru (A.V.M.); omartynova@ihna.ru (O.V.M.); 2 The Pushkin State Russian Language Institute, 117485 Moscow, Russia; 3 Psychiatric Clinical Hospital No. 1 Named after ON. Alekseeva of the Moscow City Health Department, 117152 Moscow, Russia; nataliza80@gmail.com; 4 Centre for Cognition and Decision Making, Institute for Cognitive Neuroscience, National Research University Higher School of Economics, 109548 Moscow, Russia

**Keywords:** EEG, event-related potentials, schizophrenia, fearful expressions, perception, non-congruent sounds

## Abstract

Emotional dysfunction, including flat affect and emotional perception deficits, is a specific symptom of schizophrenia disorder. We used a modified multimodal odd-ball paradigm with fearful facial expressions accompanied by congruent and non-congruent emotional vocalizations (sounds of women screaming and laughing) to investigate the impairment of emotional perception and reactions to other people’s emotions in schizophrenia. We compared subjective ratings of emotional state and event-related potentials (EPPs) in response to congruent and non-congruent stimuli in patients with schizophrenia and healthy controls. The results showed the altered multimodal perception of fearful stimuli in patients with schizophrenia. The amplitude of N50 was significantly higher for non-congruent stimuli than congruent ones in the control group and did not differ in patients. The P100 and N200 amplitudes were higher in response to non-congruent stimuli in patients than in controls, implying impaired sensory gating in schizophrenia. The observed decrease of P3a and P3b amplitudes in patients could be associated with less attention, less emotional arousal, or incorrect interpretation of emotional valence, as patients differed from healthy controls in the emotion scores of non-congruent stimuli. The difficulties in identifying the incoherence of facial and audial components of emotional expression could be significant in understanding the psychopathology of schizophrenia.

## 1. Introduction

Fear is one of six basic emotions that have considerable biological value. The emotional expressions of fear can be perceived and identified significantly more easily than other emotions and are the least affected in variable clinical populations. In particular, patients with variable mental or neurological diseases can correctly emit fearful and angry prosody of speech and fearful facial expression; however, they show impairment in the discrimination of happy expressions [1,2,3,4]. The reason for the privileged position of fearful stimuli during perception is because fear generates danger signals and leads to the mobilization of the body’s resources. In particular, the perception of fearful facial expressions is usually accompanied by heightened arousal or a negative or aversive subjective experience. It could enhance the visual processing of stimuli, activating the sympathetic nervous system [5]. The other neural basis for visual perception enhancement could include the increased work of the amygdala triggering the brain areas involved in the processes of directed attention and the visual cortex [6].

Simultaneously, perception of fearful facial expressions in real life necessarily requires multimodal analysis, including facial expression and auditory accompaniment, such as screaming, moaning, or sighing. Difficulties of multimodal perception of fearful stimulation can be associated with the ambiguous interpretation of the perceived facial expression and emotional vocalizations typical for patients with schizophrenia. According to previous studies, the perception of facial expressions plays an essential role in the clinical manifestations of schizophrenia, and the perception and expression of emotions is an important marker for assessing the severity of the emotional impairment in patients with schizophrenia [7]. In particular, deficits of emotional perception in patients with schizophrenia have previously been reported in a considerable body of literature that has demonstrated a decrease in the accuracy of recognition of emotions modulated by voice by this group of patients [8], a deficit of facial expression perception [9], and a difficulties in the understanding and awareness of other emotional expressions required for successful social interaction [10,11]. The event-related potential (ERP) studies also demonstrated the impairment of emotion perception in patients with schizophrenia [12]. For example, the significantly higher error rate in identifying non-linguistic emotional sounds in patients suffering from schizophrenia was also reported [13], as well as deficits in visual processing for all types of emotional stimuli, including fear [14].

However, the most pronounced dysfunction of emotional stimuli has been detected during the presentation of multimodal stimuli, such as facial expressions with emotions of joy, calm, and fear simultaneously with congruent or non-congruent sounds [15]. Other studies also suggest that deficits in facial information encoding extend to multimodal face–voice stimuli and that delays exist in feature extraction from multimodal face–voice stimuli in schizophrenia [16]. Moreover, the multisensory integration of emotion could be an essential element of emotional impairments in patients with schizophrenia and could involve the change of P100 and P200 amplitude for non-congruent sounds [17]. Other multimodal ERP studies have also demonstrated the involvement of P1, P2, P50, and P300 ERP components in impairments of emotional perception in subjects with schizophrenia [15,18].

At the same time, in our study, we analyzed ERP components previously detected as impaired during the auditory and visual odd-ball paradigm in patients with schizophrenia. In particular, persistent differences in N50 amplitude were previously detected between healthy subjects and co-twins with schizophrenia [19]. Other studies demonstrated reduced P3a amplitude [20] and abnormalities of the P3b wave [21] during an auditory odd-ball task in patients with schizophrenia compared with healthy controls. Our increased focus on P3a and P3b components was based on previous multimodal studies with healthy adults that also indicated the involvement of P3a and P3b during the procession of stimuli, which could be associated with orienting attention and other later cognitive and mental processes [22,23]. According to the previous findings in multimodal emotional perception, we aimed to assess the ability of patients with schizophrenia to match the compliance of emotional stimuli, presented simultaneously in visual and auditory modalities using congruent and non-congruent emotional sounds, to fearful expressions.

## 2. Methods

### 2.1. Participants

Healthy volunteers were recruited through online or institutional advertisements. In total, 20 participants of the control group (mean = 26.3, SD = 5.32, 12 female, 8 male) with no history of schizophrenia or schizoaffective disorder, intelligence quotient (IQ) less than 80, or medical illness associated with increased rates of depression, completed the study.

Twenty-two patients with schizophrenia (mean = 28.1, SD = 5.19, 10 female, 12 male) were recruited via Alekseev’s Psychiatric Clinical Hospital after a diagnostic clinical interview (ICD-10) to determine the diagnosis. Inclusion criteria included individuals with a history of first or second psychosis. The severity of symptoms was assessed in participants using the Positive and Negative Syndrome Scale (PANSS) (average meaning 93.52 ± 15.7).

All subjects were right-handed, had normal hearing levels in both ears, and their intellectual skills were within the normal range. We analyzed hearing function in healthy subjects and patients using a PDD-401 audiometer (Piston Ltd., Budapest, Hungary) to identify hearing threshold levels. None of the examined subjects had any symptoms of hearing loss.

### 2.2. Ethical Statement

The ethics board of the Institute of Higher Nervous Activity and Neurophysiology of RAS (IHNA) approved the study protocol. Electroencephalography recordings and stimuli assessment were conducted with the permission of the ethical board of Alekseev Psychiatric Clinical Hospital. All participants provided written informed consent and received monetary compensation for their participation (500 rubles). The study followed the tenets of the Declaration of Helsinki.

### 2.3. Stimuli Description

**Figure 1:**
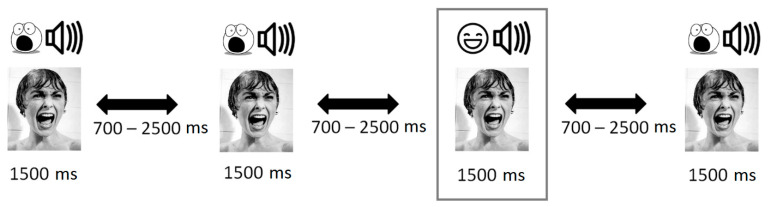
Visualization (pictures from Internet resources) of used multimodal odd-ball paradigm experimental design.

The visual stimuli consisted of centered monochrome 800-×-800-pixel.jpeg images of scared women (see Figure 1 for examples). The images of scared women were purchased from internet databases (Can Stock Photo, Fotosearch) and the International Affective Picture System (IAPS), then centered and normalized for color, brightness, contrast, background, and face size using Adobe Photoshop.

The stimuli consisted of vocalizations of women crying and laughing, which were purchased from internet databases (Sound Jay, Sound Library, Freesound, Soundboard). The raw audio files were downsampled at a rate of 44,100 Hz to mono.wav files with 32-bit resolution. The sounds were presented using the software “Presentation” (Version 20.2, Neurobehavioral Systems, Inc., Berkeley, CA, USA). All files were then normalized for root-mean-square (RMS) amplitude and modified with respect to the stimulus length using Wavelab 10.0 (Steinberg). Twenty-four original audio files (10 screaming vocalizations and 7 laughing) were submitted to pilot perceptual validation by 19 adults (students) in the pilot experiment. After the pilot study, we selected sounds with the highest rates of “pleasant” (for laughter) and “fearful” (for screaming) with the most similar rates of “arousal” and the most similar physical characteristics (duration, pitch, and loudness).

Auditory stimuli were 1500 ms (±17 ms) in the duration of sounds of women screaming and laughing, equalized by average pitch and volume.

Finally, we had three images of screaming women, three sounds of screaming vocalizations, and three sounds of laughing vocalizations. After a pilot study, we concluded that different images with their own vocalizations induced the most pronounced emotional response and excluded addictive effects, so for each image of screaming women we selected the appropriate vocalization

The standard (congruent) stimulus was the simultaneously presented image and sound of a screaming woman 1500 ms long. The deviant (non-congruent) stimulus simultaneously presented an image of a screaming (frightened) woman and the sound of her laughter 1500 ms long. We used three types of stimuli to exclude the addictive effect.

### 2.4. Procedure

The participants sat in front of the screen. The instruction was to listen to a sound and look at the screen. The stimuli were presented via a pair of TDH-39 headphones. Two types of multimodal visual–voice stimuli with semantical incoherence were presented: the same emotional expression and an incoherent emotional expression. An odd-ball passive paradigm was used with EEG event-related potentials (80% coherent and 20% incoherent). We made three trials of two conditions (coherent/incoherent) with three parameters (three kinds of sounds and images for the condition to avoid an addictive effect). The stimuli were presented in random order. Participants were instructed to “view and listen to stimulation, don’t close eyes and try not to think about something special.” Visual stimuli were presented for 1500 ms centrally on a 17″ LCD monitor with a 60/120 Hz screen refresh rate; participants sat 80 cm away from the screen. The interstimulus interval randomly varied from 1000 ms to 2500 ms.

### 2.5. Self-Assessment

Immediately before the main experimental series (when the EEG helmet was placed on the subject’s head), we asked participants to assess their states on a scale from 1 to 9: “Sad,” “Happy,” “Angry,” “Scared,” and “Relaxed.”

### 2.6. Subjective Assessment of Stimuli

After the subject listened to the whole set, they were presented with the image of a sad face and the sound of crying (congruent stimulus) on the same monitor with the scales. They were then required to evaluate the stimulus on scales of 1 to 9 for each of the following terms: “sad,” “happy,” “angry,” “fearful,” and “calm.” After filling in the scales, four incongruent stimuli were presented, which the subject evaluated on the same scales. If difficulties arose in understanding the task, the subject was assisted by the researcher.

### 2.7. EEG Registration

EEG was acquired using a 19-channel EEG amplifier Encephalan with the recording of polygraphic channels (Poly4, Medicom MTD, Taganrog, Russian Federation). The sampling rate was 250 Hz. The amplifier bandpass filter was nominally set to 0.05–70 Hz. AgCl electrodes (Fp1, Fp2, F7, F3, Fz, F4, F8, T3, C3, Cz, C4, T4, T5, P3, Pz, P4, T6, O1, and O2) were placed according to the International 10–20 system. The electrodes placed on the left and right mastoids served as joint references under unipolar montage. The vertical oculogram was recorded with AgCl cup electrodes placed 1 cm above and below the left eye, and the horizontal EOG was acquired by electrodes placed 1 cm lateral from the outer canthi of both eyes. The electrode impedances were kept below 10 kΩ. The EEG fragments did not contain any epileptiform activity (exclusion criteria).

### 2.8. Data Analysis

The EEG data were processed using a 0.5–30 Hz bandpass filter (finite impulse response filter). The 50 Hz power frequency noise was subject to notch processing. The reference electrode was changed to a global brain average reference. Artefacts due to eye movement were excluded by independent ICA component analysis by an ICA-based algorithm with the EEGLAB plugin for MatLab 7.11.0 (MathworkInc., Natick, MA, United States). Muscle artefacts were cut out through manual data inspection. Finally, 111.3 ± 5.3 artefact-free EEG epochs of congruent stimuli and 26.8 ± 2.8 epochs of non-congruent stimuli in the control group and 112.5 ± 5.8 and 27.1 ± 2.9 epochs in the group of patients were applied for further data analysis.

The segments of interest were located within the whole data file and extracted with encephalograph proprietary software Encephalan EEGR 19/26. For the ERP analyses, the EEG data were analyzed and processed using EEGLAB 14.1.1b, which is a neural electrophysiological analysis tool based on MATLAB (MathWorks, Natick, MA, USA). The EEG was segmented from 100 ms prior to initiation to 800 ms after the stimulus onset. Amplitude and latency of each component were calculated for each participant in both groups separately for statistical analysis.

### 2.9. Event-Related Potential (ERP) Analysis

Amplitude and latency of each component were calculated for each participant in both groups separately for statistical analysis. For the ERP analyses, the EEG data were analyzed and processed using EEGLAB 14.1.1b, which is a neural electrophysiological analysis tool based on MATLAB (MathWorks, Natick, MA, USA). The EEG data were processed using a 1.6–30 Hz bandpass filter (finite impulse response filter). The 50 Hz power frequency noise was subject to notch processing. The reference electrode was changed to a global brain average reference. Artefacts due to eye movement were excluded by independent component analysis. The EEG was segmented from 100 ms prior to initiation to 800 ms after the stimulus onset. In this study, the amplitudes and latencies of N50, P100, N200, P3a, and P3b were measured and analyzed. Based on the topographical distribution of the grand-averaged ERP activity, the following sets of electrodes for each component were chosen: Fz, F3, F4, Сz, C3, C4 were selected for the analysis of N50 (30–90 ms) and P100 components (90–160 ms); N200 components (160–250 ms), were analyzed at the Fz, F3, F4, Cz, C3, C4, Pz, P3, P4, O1, O2 electrode sites; P3a components (220–300 ms) were analyzed at the F3, Fz, F4, Cz, C3, C4 electrode sites; and P3b components (250–500 ms) were analyzed at the Cz, C3, C4, Pz, P3, P4, O1, O2 electrode sites. The individual maximum peak amplitude was extracted for each component for each subject using MATLAB (MathWorks, Natick, MA, United States). In the cases where subjects had no well-defined peak (as in the case of P3a in group of patients) we used a time window of 20 ms around maximum to extract averaged amplitude.

### 2.10. Statistical Analysis

Statistical analysis was carried out with STATISTICA 13 (TIBCO Software, Palo Alto, CA, USA). Differences between groups, differences in amplitudes, and latencies of ERP components, as well as subjective ratings, were assessed using a nonparametric Mann–Whitney u-test followed by post-hoc comparison (Bonferroni, *p* < 0.05). The repeated-measures ANOVA was used to assess separately for the attraction of two effects: condition (standard stimulus and deviant stimulus) * group.

The correlation analysis between subjective ratings and ERP data was calculated using Spearman rank correlations with Bonferroni correction (*p* < 0.05) separately for each group of subjects. All reported values of Spearman r-test had a power more than 80%. The inside group differences between amplitude and latency of ERP components of congruent and non-congruent stimuli for each ERP component were calculated using non-parametrical Wilcoxon rank tests.

## 3. Results

### 3.1. Differences in Subjective Ratings of Stimuli

Groups did not differ in their individual ratings of self-assessments for the terms “Sad,” “Happy,” “Angry,” “Scared,” and “Relaxed.” Non-congruent stimuli were assessed as being happier by patients than by subjects of the control group (z = 2.6, *p* = 0.008); controls had 2.2 ± 1.8 scores on the scale “happy,” assessing non-congruent stimuli, versus the 5.3 ± 2.1 scores in patients.

### 3.2. Group Differences in ERP Components to Congruent and Non-Congruent Stimuli

**Figure 2:**
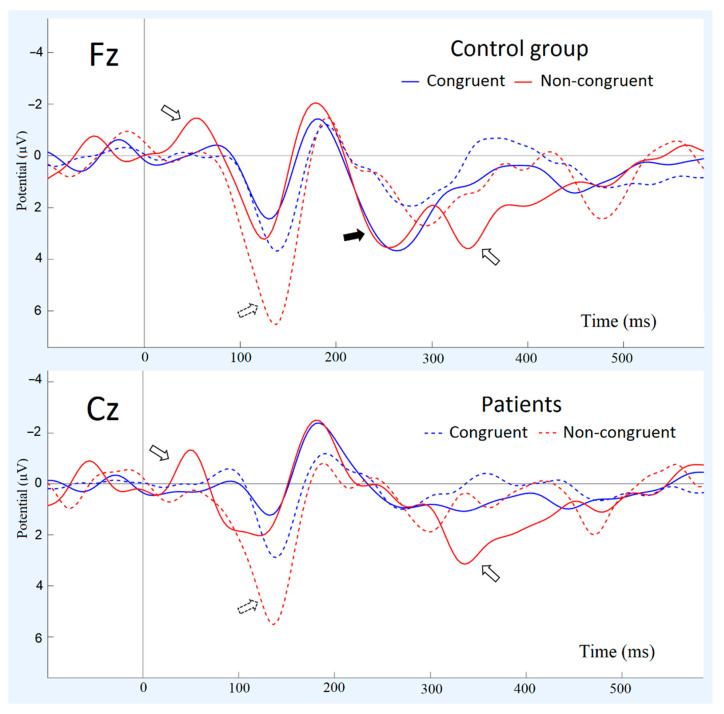
Event–related potential (ERPs) for congruent and non–congruent stimuli in two electrode sites (Fz, Cz) for two groups (patients with schizophrenia and healthy controls): Potential (µV). Arrows indicate significant group differences: black contour arrow means that healthy subjects had differences between congruent and non–congruent, but patients did not; black dotted arrow means that patients had differences between congruent and non–congruent stimuli and control group did not; black arrow means that there is a significant group difference in component’s amplitude but no differences between stimuli.

Patients had longer latency of N50 for both congruent (Mann–Whitney U Test; z = 2.8, *p* = 0.004) and non-congruent stimuli (z = 2.6, *p* = 0.008), lower amplitude of N50 for non-congruent stimuli (z = −2.9, *p* = 0.001), larger amplitude of P100 for non-congruent stimuli (z = 2.1, *p* = 0.03), larger amplitude of N200 (z = 2.4, *p* = 0.02), lower amplitude of P3a for both congruent (z = -2.2, *p* = 0.03) and non-congruent stimuli (z = −2.3, *p* = 0.02), and lower amplitude of P3b for non-congruent stimuli (z = −2.4, *p* = 0.01) (see Figure 2, Table 1).

**Figure 3:**
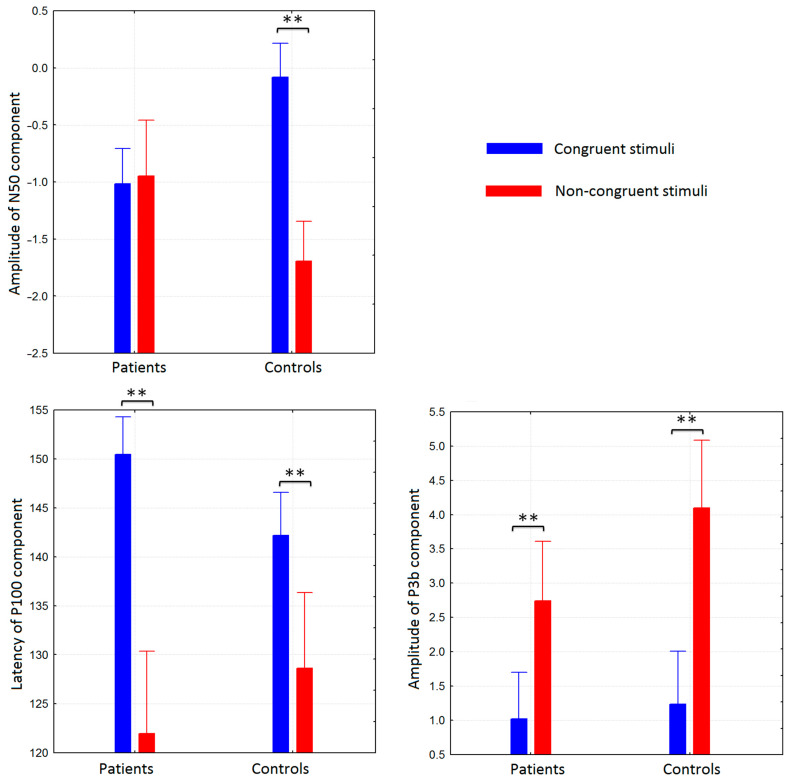
Values of amplitudes and latencies of N50, P100, and p3b ERP components for two groups (patients with schizophrenia and healthy controls) averaged as described in section “Event–related potential (ERP) analysis”. The significant differences (*p* < 0,01) were marked by “**”.

Figure 3 also illustrates the differences in ERP components. The amplitude of N50 was significantly higher for non-congruent stimuli than for congruent stimuli only in the control group (Repeated measures ANOVA stimuli*group F(1,40) = 6.3628, *p* = 0.008). Both groups demonstrated longer latency of P100 for congruent stimuli compared to non-congruent stimuli (stimuli effect; F(1,40) = 8.8266, *p* = 0.00729). Both groups had lower amplitudes of P3b for congruent stimuli compared to non-congruent stimuli (stimuli effect; F(1,40) = 15.857, *p* = 0.0007).

### 3.3. Correlations between ERP and Subjective Ratings and PANSS

**Figure 4:**
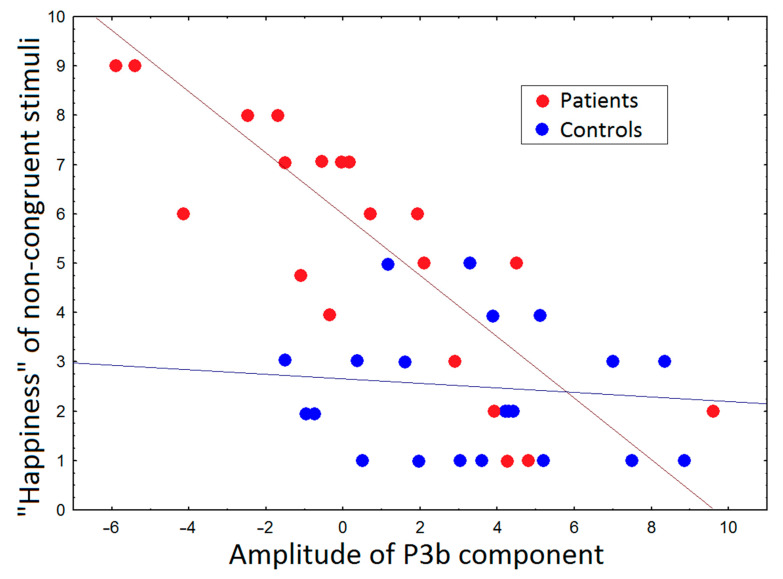
Scatter–plot of individual values demonstrated significant correlation between the amplitude of P3b component for “happiness” of non–congruent stimuli in group of patients and healthy control.

The amplitude of P100 for both congruent (r = 0.65, *p* = 0.01) and non-congruent stimuli (r = 0.59, *p* = 0.03) correlated with PANSS scores. Scores of self-assessment by scale “calm” in the group of patients negatively correlated with a latency of P100 (r = −0.73, *p* = 0.008), N200 (r = −0.83, *p* = 0.005), and P3a (r = −0.83, *p* = 0.006) components for congruent stimuli. The amplitude of P3b for non-congruent stimuli negatively correlated with the happiness of stimuli in the group of patients (r = −0.81, *p* = 0.007) and did not reach significance in healthy controls (z = −0.52, *p* = 0.06) (Figure 4).

## 4. Discussion

We identified that the amplitude of N50 was significantly higher for non-congruent stimuli only in subjects. In contrast, patients had reduced N50 amplitude and longer latency for both congruent and non-congruent stimuli. As was shown previously, the early negative component around 50 ms (N50) was generated in the auditory cortex as a response to rare stimuli and was associated with the encoding of multimodal information [7]. Other studies reported that the appearance of N50 could be modulated by percept repetition [24] and was associated with the perception of angry, happy, or sad facial expressions. Therefore, the difference between congruent and non-congruent stimuli, which was found in healthy controls and reduced in patients with schizophrenia, could be explained by, first, early sensory abnormalities during emotional recognition [25], and second, by the deficit of implicit perceptual memory, which modulated the early processing of multimodal stimuli [26].

The larger amplitude of P100 and N200 components correlated with PANSS scores, which was found in a group of patients, could be explained by the well-known sensory-gating effect usually reduced in patients with schizophrenia. Previous studies showed that the amplitude of early components of multimodal ERPs should typically be decreased compared to ERP for a single modality [27]. The origin for the decrease of ERP components’ amplitude could be explained by the sensory-gating phenomenon revealed in multisensory studies [28]. We hypothesized that the sensory-gating effect was the reason for the lower amplitude of the components N200 and P100 in the subjects of the control group, and the lack of sensory gating provided the absence of amplitude decrease in patients with schizophrenia [29]. The previous findings could explain the origin of these group differences between patients and the control group, demonstrating that multisensory integration requires the connections between unisensory and polysensory brain regions, which could be impaired in patients with schizophrenia [30].

The difference in the assessment of congruent and non-congruent stimuli, which was significantly higher in the control-group subjects and more stable in the group of patients, correlated with a difference in the P100 amplitude for congruent and non-congruent stimuli in the left-temporal area. The P100 amplitude was shown to reflect general primary visual analyses in the striate and cortex [31]. It was also sensitive to the perception of emotionally charged visual stimuli [32]. In our study, only healthy subjects showed a significant increase in the amplitude of P100 to non-congruent stimuli compared to congruent. The lower amplitudes of P100 with no differences between congruent and non-congruent stimuli in patients with schizophrenia were consistent with previous findings, which demonstrated a decrease of the amplitude of P100 in patients with schizophrenia during the processing of emotional faces compared to healthy peers [33]. The reductions in P100 amplitude to emotional compared to neutral faces were revealed in patients with low-to-medium shyness [34] and children with autism spectrum disorder (ASD) [35]. Other studies also revealed that patients with schizophrenia demonstrated P100 changes during multimodal stimulation [16]. The absence of P100 changes in patients with schizophrenia could be explained by the impaired processing of emotional stimuli that were not modulated by the emotional identity of faces compared to healthy controls [36].

The deficit of implicit perceptual memory and attention in a group of patients was also revealed for the late ERP components. In particular, we found that patients had lower amplitude of P3a for both congruent and non-congruent stimuli. According to the previous findings, the reduction of P3a could be described as a reliable biomarker of schizophrenia and was associated with deficits in verbal memory and attentional switching, reflecting dysfunctions in the temporal and frontal systems [20,37]. At the same time, the reduction of P3a in patients with schizophrenia could be associated with less emotional arousal during emotional perception or incorrect interpretation of emotional valence. In particular, this ERP component was reported to be reduced in the early stages of a psychotic illness marked by disturbances in perception and affect [38,39]. The enhanced P3a amplitudes in our study during the perception of the screaming multimodal stimuli is in accordance with previous findings regarding the relation of P3a with the unpleasantness of pictures, so the P3a amplitude could be sensitive to the arousal value of unpleasant stimuli in subjects of the control group [40].

The perception of non-congruent stimuli was accompanied by a higher rate of patients’ self-assessment scores by scale “happy”. In particular, the scared face accompanied with the sound of laughter was assessed as being happy more often by patients than by healthy controls. Moreover, these subjective rates were negatively associated with the amplitude of P3b, which was higher for non-congruent stimuli in both groups of subjects but achieved significance only in patients with schizophrenia. According to previous findings, the amplitude of P3b could be related to expression categorization [8,9,10,11,12,13,14,15,16,17,18,19,20,21,22,23,24,25,26,27,28,29,30,31,32,33]; moreover, the P3b component was shown to be modulated by the emotional arousal and valence of the non-congruent pictures [41]. At the same time, in our study, the picture demonstrated fearful expression. As we revealed, the analysis of stimuli and assessing patients’ happiness in healthy controls was based predominantly on visual stimuli, unlike the group of patients. Our results demonstrated that audio-visual cross-modal processing in patients with schizophrenia was predominantly modulated more by the affective arousal and valence of non-congruent sounds than by pictures. Thus, impairments in neural synchrony could be related to sensory demands and the processing of multimodal information [42,43] and were accompanied by ambiguous or incorrect interpretation of the perceived facial expression and emotional vocalizations.

## 5. Conclusions

Patients with schizophrenia demonstrated altered ERPs reflecting the impaired multimodal perception of fearful stimuli compared to healthy controls. The differences were revealed both for the early and late stages of stimuli processing. Compared to healthy controls, patients had reduced amplitudes of N50 for non-congruent stimuli, which could be associated with sensory–perception abnormalities and the deficit of implicit perceptual memory during emotional multimodal recognition. Patients with schizophrenia also had higher amplitudes of P100 and N200 components, explained by impaired sensory gating. Moreover, P3a and P3b components’ reduced amplitude in patients could be associated with attention deficiency, less emotional arousal, or incorrect interpretation of emotional valence. The latter assumption was supported by a significant difference in the subjective ratings of emotions in the case of non-congruent stimuli in schizophrenia patients compared with healthy controls.

## Figures and Tables

**Table 1:** The amplitudes and latencies of each ERP component for congruent and non-congruent stimuli and for each group of subjects (patients and controls).

		N50	P100	N200	P3a	P3b
Control group						
Congruent stimuli	amplitude	−0.1 ± 0.4	1.9 ± 0.5	−3.9 ± 0.7	2.7 ± 0.7	1.2 ± 0.5
	latency	62 ± 9.7	142.2 ± 22	188.8 ± 31	266.0 ± 32	337.2 ± 35
non-congruent stimuli	amplitude	−1.6 ± 0.3	3.8 ± 0.6	−4.3 ± 1.0	2.6 ± 0.6	4.1 ± 1.2
	latency	49.6 ± 8.8	128.6 ± 29	200.2 ± 37	273.1 ± 44	312.9 ± 49
Patients with schizophrenia						
Congruent stimuli	amplitude	−0.2 ± 0.5	4.6 ± 0.6	−3.3 ± 0.4	0.7 ± 0.4	1.0 ± 0.7
	latency	88.8 ± 12	152.5 ± 45	214.9 ± 39	274.8 ± 43	371.8 ± 58
non-congruent stimuli	amplitude	−0.2 ± 0.4	7.6 ± 1.3	−2.8 ± 1.1	0.5 ± 1.0	2.7 ± 0.8
	latency	66.2 ± 15	122.9 ± 27	207.1 ± 36	260.6 ± 45	356.1 ± 55

## Data Availability

The data that support the findings of this study are available on request from the corresponding author.
